# A Novel Class of Ribosome Modulating Agents Exploits Cancer Ribosome Heterogeneity to Selectively Target the CMS2 Subtype of Colorectal Cancer

**DOI:** 10.1158/2767-9764.CRC-22-0469

**Published:** 2023-06-05

**Authors:** Esteban Terzo, Shruti A. Apte, Simran Padhye, Saleh Rashed, Wesley Austin, Michael Caponegro, Anupama Reddy, Shuhao Shi, Christy Wang, Roger B. Clark, David Sidransky, Vijay Modur, Vasudeo Badarinarayana

**Affiliations:** 1 Eloxx Pharmaceuticals, Watertown, New York.; 2 Vindhya Data Science, Data Science, Morrisville, North Carolina.; 3 Johns Hopkins University School of Medicine, The Sidney Kimmel Comprehensive Cancer Center, Baltimore, Maryland.

## Abstract

Ribosomes in cancer cells accumulate numerous patient-specific structural and functional modifications that facilitate tumor progression by modifying protein translation. We have taken a unique synthetic chemistry approach to generate novel macrolides, Ribosome modulating agents (RMA), that are proposed to act distal to catalytic sites and exploit cancer ribosome heterogeneity. The RMA ZKN-157 shows two levels of selectivity: (i) selective translation inhibition of a subset of proteins enriched for components of the ribosome and protein translation machinery that are upregulated by MYC; and (ii) selective inhibition of proliferation of a subset of colorectal cancer cell lines. Mechanistically, the selective ribosome targeting in sensitive cells triggered cell-cycle arrest and apoptosis. Consequently, in colorectal cancer, sensitivity to ZKN-157 in cell lines and patient-derived organoids was restricted to the consensus molecular subtype 2 (CMS2) subtype that is distinguished by high MYC and WNT pathway activity. ZKN-157 showed efficacy as single agent and, the potency and efficacy of ZKN-157 synergized with clinically approved DNA-intercalating agents which have previously been shown to inhibit ribogenesis as well. ZKN-157 thus represents a new class of ribosome modulators that display cancer selectivity through specific ribosome inhibition in the CMS2 subtype of colorectal cancer potentially targeting MYC-driven addiction to high protein translation.

**Significance:**

This study demonstrates that ribosome heterogeneity in cancer can be exploited to develop selective ribogenesis inhibitors. The colorectal cancer CMS2 subtype, with a high unmet need for therapeutics, shows vulnerability to our novel selective ribosome modulator. The mechanism suggests that other cancer subtypes with high MYC activation could also be targeted.

## Introduction

Over the last 20 years, a growing body of evidence indicates that ribosome abundance, composition, stoichiometry, and function can vary significantly between developmental states, tissues, cell types and even at different subcellular locations in the same cell (1, 2). Tissue- or disease-specific expression of ribosomal protein genes (RPG), rRNA variants, as well as differential chemical modification of rRNA bases contribute to ribosome heterogeneity. This ribosome heterogeneity can alter the structure, processivity, and transcript selectivity of ribosomes and provides another mechanism for selectively regulating protein expression in response to various stimuli or cell states (1, 2).

In cancer, mutations, and copy-number alterations in RPGs, dysregulated expression of small nucleolar RNAs (snoRNA) that control chemical modification of specific rRNA residues, and dysregulation of various steps involved in ribogenesis has been shown to facilitate oncogenesis (3–6). These cancer-specific alterations in ribosomes leading to addiction of cancer cells to altered protein translation patterns provide an opportunity to selectively target cancer ribosomes while potentially sparing other cell types.

Because of the critical function played by ribosomes in oncogenesis, targeting the cancer ribosome has been a focus of earlier drug development efforts (7). Many molecules have been identified that target the different catalytic steps occurring in the ribosome during protein synthesis (8). However, these steps are not just required for oncogenesis, but also required for normal protein synthesis leading to poor selectivity of these agents and resultant narrow therapeutic window.

Traditional macrolide antibiotics are allosteric inhibitors that bind the bacterial ribosome in the nascent peptide exit tunnel (NPET) to selectively inhibit translation of proteins enriched for positively charged amino acids resulting in a bacteriostatic effect (9). While the macrolide binding site between prokaryotes and eukaryotes is generally conserved, macrolide antibiotics do not bind or modulate eukaryotic ribosome function nor are used in cancer therapy. This is because of a small difference, the adenosine at position 2058 of the 23S rRNA (*Escherichia coli* numbering), which in eukaryotes is replaced with guanine (9, 10) and consequently precludes binding of macrolide antibiotics to eukaryotic ribosomes. Because small changes in rRNA sequence can drive large differences in selectivity of macrolides for the eukaryotic ribosome, we have synthesized novel macrolides called ribosome modulating agents (RMA) using our unique chemistry (11) that can explore an alternative chemical space with potential to target the eukaryotic ribosome.

We present data demonstrating that ZKN-157, a representative RMA, selectively inhibits protein translation in a subset of cancer cell lines. Consequently, treatment of sensitive cells lines with ZKN-157 triggered cell-cycle arrest and apoptosis. We identified that the proteins impacted by ZKN-157 in sensitive cancer cells are enriched for proteins containing high density of positively charged regions. This pattern of protein translation inhibition by protein charge characteristics also enriched for components of the ribosome and protein translation machinery that are regulated by MYC. Further evaluation of ZKN-157 across a panel of colorectal cancer–derived cell lines and patient-derived organoids identified that the consensus molecular subtype 2 (CMS2; refs. 12, 13) of colorectal cancer characterized by high MYC and WNT pathway activation is particularly sensitive to ZKN-157. Finally, we show that the potency and efficacy of ZKN-157 synergizes with clinically used DNA-intercalating agents that are known to inhibit ribogenesis (14). We have invented a new class of molecules that target a critical pathway in cancer to selectively induce cell-cycle arrest and apoptosis in cancer cells. On the basis of this novel mechanism, our tailored molecules can be used as monotherapy or combined with approved chemotherapeutics in a rational manner to target specific types of cancer.

## Materials and Methods

### Cell Culture

Cell lines were acquired from ATCC: SW1417, CCL-238, RRID: CVCL_1717; COLO320DM, CCL-220, RRID: CVCL_0219; SW403, CCL-320, RRID:CVCL_0545; and SW948, CCL-237, RRID:CVCL_0632. COLO320DM were cultured in RPMI Media (ATCC 30-2001). SW1417, SW403, and SW948 were cultured in DMEM (GIBCO 10569-010). Cell lines were maintained in humidified 37°C incubators with 5% CO_2_. All media were formulated using 10% FBS and 1% penicillin/streptomycin. Assay-specific media formulations are described in their corresponding sections. All cell lines were tested for *Mycoplasma* contamination using the Myco Alert Assay Control Set (Lonza LT07-518) following the manufacturer's instructions and used only if results were negative. SW1417, COLO320DM, and SW403 cell lines were authenticated by Labcorp. For all experiments in this study, all cell lines were grown and expanded for a maximum of 15 passages from their thawing.

### Proliferation Assay

Cells were resuspended in 40 μL of medium, seeded in 384-well flat transparent bottom tissue culture–treated plates (Greiner 781098) at the following densities: COLO320DM (400/well), SW1417, SW403 (600/well), and 1,000/well (SW948) and incubated for 24 hours. All treatments were performed using the D300e digital liquid dispenser (TECAN). ZKN-157 was dosed as a single agent starting at 60 μmol/L in both an 8-point 3-fold serial dilution, or a 10-point 2-fold serial dilution (combinatorial treatments). Mitoxantrone (Selleckchem S2485), doxorubicin (Selleckchem S1208) or actinomycin-D (Sigma-Aldrich SBR00013) were dosed in a 10-point 3-fold dilution series. DMSO's limit for all treatments was 0.2%. Treatment timepoints for COLO320DM (48 hours) and for SW1417, SW403, and SW948 (144 hours) were selected to span at least two cell doublings. ATP levels were measured as readout for cell viability using CellTiter-Glo 2.0 reagent (Promega G9243) for all timepoints, including day 0 (untreated cells). Luminescence reading was performed using Cytation5 Multi-Mode plate reader (Agilent). Minimum response percentage values were calculated using (IF) logical test: = IF(T>T0,100*(T − T0)/(C − T0),100*(T − T0)/T0). Key: T: Drug treatment; T0: Drug treatment at time zero; C: Vehicle treatment. To ensure that GI_50_ in each cell line is measured at the same number of cell doublings (in this case two cell doublings), the GI_50_ for all cell lines were evaluated at timepoints based on their specific doubling time. This is the recommended protocol from the NCI (https://dtp.cancer.gov/discovery_development/nci-60/methodology.htm).

Drug synergy was analyzed using the Bliss independence (BI) principle. BI scores were calculated from averaged values (two replicates/plate/tested dose as single agent or in combination) using the following formula: Observed − Expected = BI score. BI scores were defined as follows: antagonistic < −0.14; −0.14 < additive < 0.14; and synergistic > 0.14. The first column (ZKN-157) and bottom row (actinomycin D, doxorubicin, or mitoxantrone) of each matrix were used for single-agent treatments.

### Metabolic Labeling

Cells were seeded at a density of 7 × 10^5^ (SW1417) and 5 × 10^5^ (COLO320DM) in a 6-well plate for 48 and 24 hours, respectively. Cells were treated with DMSO and 40 μmol/L of ZKN-157 for 24 hours and 50 μg/mL of cycloheximide (CHX; Sigma) for 2 hours prior to incubation in methionine free (-Met) media. L-Azidohomoalanine (AHA) incorporation and Click-iT reaction were performed as in ref. 15. Specific modifications are detailed in the Supplementary Data.

### pSILAC

DMEM for SILAC (Gibco) was supplemented with dialyzed FBS (Gibco). The light media was supplemented with L-Lysine-2HCl for SILAC (Thermo Fisher Scientific) and L-Arginine-HCl for SILAC (Gibco). The heavy media was supplemented with L-Lysine-2HCl, ^13^C_6_, ^15^N_2_ for SILAC (Thermo Fisher Scientific) and L-Arginine-HCl, ^13^C6, ^15^N4 for SILAC (Thermo Fisher Scientific). SW1417 cells were seeded at a density of 2 × 10^6^ cells per 60 mm dish in light media (+Light Arg/Lys) for 24 hours. Cells were treated with DMSO and ZKN-157 (20 μmol/L) for 1 hour in DMEM for SILAC with dialyzed FBS (-Arg/Lys), followed by a wash with warm heavy media (+Heavy Arg/Lys). Cells in both conditions were incubated in +Heavy Arg/Lys media for a total of 6 and 24 hours. Subsequently, cells were scrapped off, collected in a 1.5 mL tube, and centrifuged at 500 × *g* for 5 minutes. Cell pellets were washed twice with PBS, centrifuged at 500 × *g* for 5 minutes, and stored at −20°C until ready to be processed for mass spectrometry.

### Mass Spectrometry

Cell pellets were lysed in 5% SDS, 50 mmol/L triethylammonium bicarbonate (TEAB) pH 8.5, and protein concentration was determined using the BCA protein assay. Samples were reduced in 10 mmol/L dithiothreitol for 30 minutes at 37°C followed by alkylation in 20 mmol/L iodoacetamide for 20 minutes at room temperature. Samples were acidified and digested on an S‐Trap spin column (Profiti) by addition of trypsin at 1:20 for 3 hours at 47°C. Peptides were eluted in 50 mmol/L TEAB pH 8.0, 0.2% formic acid and 50% acetonitrile, respectively.

A total of 2 μg of protein per sample were used for LC/MS-MS analysis. The raw mass spectrometric data were processed in MaxQuant (version 2.0.3.0) for peptide and protein identification. More detailed protocol in the Supplementary Data.

### Immunoblotting

Assay performed as in ref. 15. Specific modifications are detailed in the Supplementary Data.

### Cell-cycle Analysis

SW1417 cells were plated at a density of 0.1 × 10^6^ cells/well in a 12-well plate, and COLO320DM cells were plated at a density of 1 × 10^6^ cells/well in a 6-well plate. After 24 hours, cells were treated with DMSO or ZKN-157 at various concentrations for 48 hours. Following treatment, cell pellets were washed twice with 1X PBS, and then fixed with Fixation buffer (BioLegend) for 15 minutes at room temperature. The pellets were then washed with Staining buffer (BioLegend) and then permeabilized with 1X Click-iT permeabilization and wash reagent (Thermo Fisher Scientific) for 15 minutes at room temperature. The cells were then stained with DNA-specific dye FxCycle Violet Stain (Invitrogen) for 30 minutes and analyzed using the BD FACSCelesta Cell Analyzer (flow cytometer).

### Organoid Culture

Colorectal cancer–derived (CMS2) organoids (14) and a normal organoid from the Hubrecht Organoid Technology (HUB) were thawed, expanded, and screened following HUB's guidelines and using formulated media internally established. Colorectal cancer–derived organoid cultures and the normal organoid culture were propagated and screened in colon tumor medium (CTM) and colon surrogate medium (CSM), respectively. CTM is routinely used for the culture and screening of colon cancer organoids. CSM contains NGS-Wnt and is routinely used for the culture and screening of colon organoids derived from healthy tissue. Organoid culture composition table, organoid handling, and treatment conditions are detailed in the Supplementary Data.

### Characterization Data for ZKN-157 Citric Acid Salt

^1^H NMR (400 MHz, CD_3_OD) *δ* 7.24 (t, *J* = 7.8 Hz, 1H) 6.88 – 6.96 (m, 2H) 6.85 (dd, *J* = 8.2, 2.0 Hz, 1H) 4.56 (br d, *J* = 13.6 Hz, 1H) 4.46 (d, *J* = 7.2 Hz, 1H) 4.20 (d, *J* = 10.0 Hz, 1H) 3.79 (s, 3H) 3.67 – 3.75 (m, 1H) 3.62 (br s, 2H) 3.43 (dd, *J* = 10.4, 7.2 Hz, 1 H) 3.33 (br s, 1H) 3.23 – 3.29 (m, 1H) 3.11 – 3.20 (m, 1H) 2.94 – 3.07 (m, 5H) 2.87 (br s, 3H) 2.69 – 2.82 (m, 11H) 2.19 – 2.47 (m, 4H) 2.13 (br dd, *J* = 12.8, 8.0 Hz, 1H) 2.01 (dt, *J* = 10.6, 2.0 Hz, 1H) 1.90 – 1.97 (m, 1H) 1.80 (br d, *J* = 13.2 Hz, 2H) 1.28 – 1.56 (m, 17 H) 1.06 (br d, *J* = 6.6 Hz, 3H). MS (ESI) *m/z* 690.5 (M+H). 99.7% purity by HPLC (UV @ 220 nmol/L). Yellow solid.

Quality control Hydrogen Nuclear Magnetic Resonance (HNMR), LC/MS, and High Performance Liquid Chromatography (HPLC) data for ZKN-157 are provided as a Supplementary Data.

### Software Analysis

GraphPad Prism 9 (RRID:SCR_002798) was used to plot percent response dose curves and bar graphs, calculate GI_50_ and LD_50_ values, and plot bar graphs for protein intensities. Image Studio 5.2 (LI-COR, RRID:SCR_015795) was used to acquire images and quantitate intensities from Smear and Western blot membranes. Spotfire TIBCO 12.0.0.223 (RRID:SCR_008858) was used to plot volcano plots, bar graphs, pie chart, and scatter plot. Fiji 2.6.0 (RRID:SCR_002285) image processing package software was used to analyze in-cell metabolic labeling (AHA) assay.

### Data Availability Statement

The data generated in this study are available within the article and its Supplementary Data.

## Results

### ZKN-157 Selectively Inhibits Protein Translation in a Sensitive but not in an Insensitive Colorectal Cancer Cell Line

**FIGURE 1:**
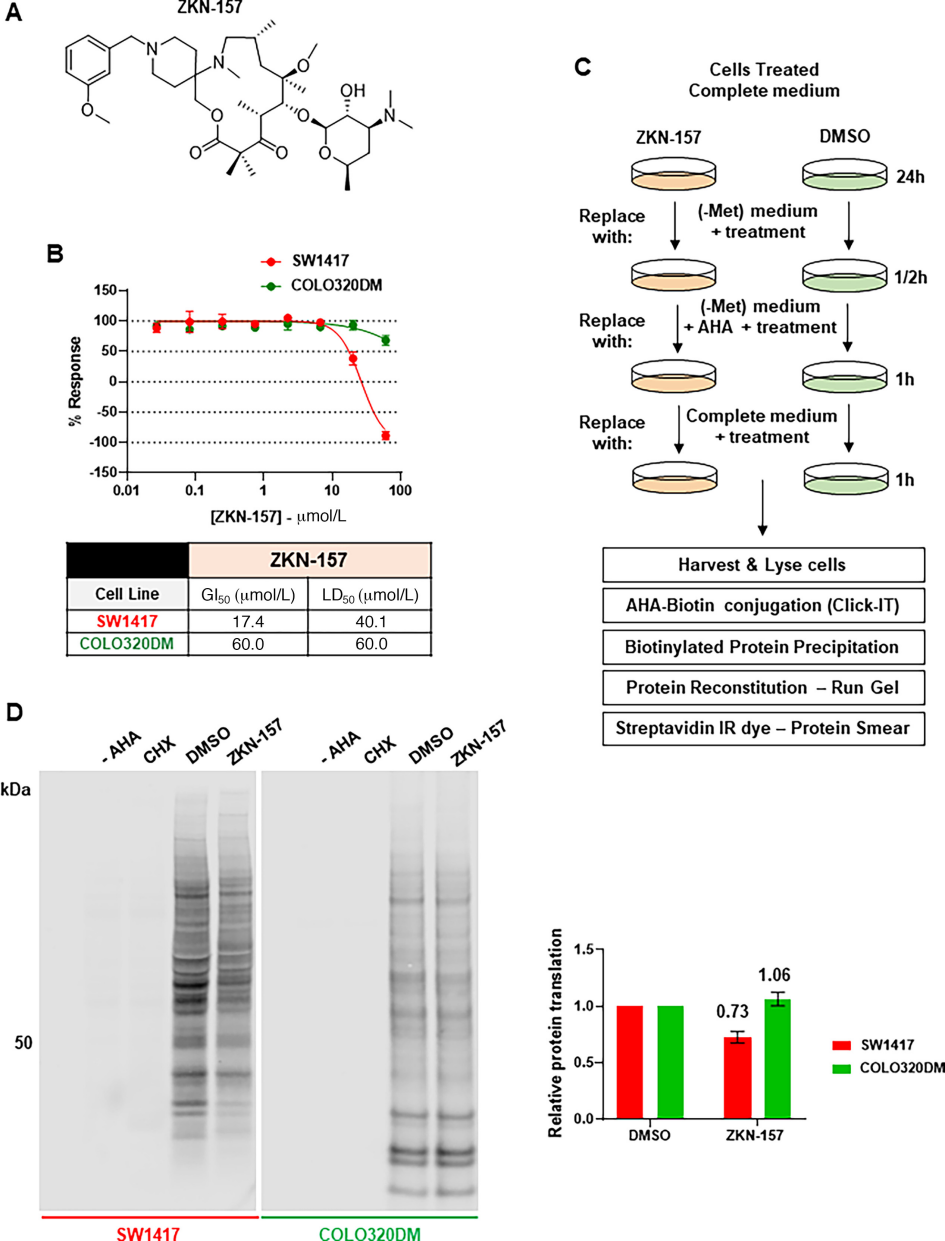
ZKN-157 selectively inhibits growth and protein translation in a sensitive colorectal cancer cell line. **A,** Chemical structure of ZKN-157. **B,** Percent response curves showing the effect of ZKN-157 treatment in COLO320DM and SW1417 cells. Curves are representative of three independent biological experiments. Error bars are SDs of two technical replicates. Table shows GI_50_ and LD50 values **C,** Cartoon showing the metabolic labeling assay workflow. **D,** Representative Western blot membranes of two independent biological experiments showing: -AHA (no AHA reagent added to lysate), CHX (positive control for translation inhibition), DMSO (negative control), and ZKN-157 treatment (40 μmol/L). Equal protein amount for all samples were used for click chemistry reaction. Samples resuspended in equal volume were loaded on gel. Intensity from densitometry of ZKN-157–treated samples was normalized to those of DMSO samples to calculate relative protein translation. Bar graph shows normalized values from two independent experiments. Error bars are SDs.

We evaluated the impact of ZKN-157 (Fig. 1A) on proliferation of two colorectal cancer cell lines (SW1417 and COLO320DM). Treatment with ZKN-157 shows a growth inhibitory effect on SW1417 cells while displaying a minimal antiproliferative activity in COLO320DM cells (Fig. 1B). To determine whether the antiproliferative activity of ZKN-157 is due to impact on protein translation, we conducted a metabolic labeling study using L-Azidohomoalanine (AHA), which is incorporated into newly synthesized proteins in place of methionine (Met; Fig. 1C). L-AHA incorporated into newly synthesized proteins was biotinylated and detected by Western blot analysis. Treatment of SW1417 with ZKN-157 for 24 hours resulted in a 27% decrease in AHA incorporation into newly synthesized proteins. In contrast, ZKN-157 treatment had no significant impact on AHA incorporation in COLO320DM cells (Fig. 1D). These results contrast with the global effect observed after CHX treatment, which completely inhibited new protein synthesis in both cell lines. This decrease in new protein synthesis was confirmed in a plate reader–based assay that uses fluorescence to quantitate AHA incorporation into newly synthesized proteins. In SW1417 cells, ZKN-157 inhibited new protein synthesis in a dose-dependent manner (Supplementary Fig. S1A and S1B). Taken together, these data indicate that ZKN-157 selectively inhibited protein translation in SW1417 cells and that this partial inhibition of protein translation is associated with growth inhibition.

### ZKN-157 Selectively Inhibits Translation of a Subset of Proteins

**FIGURE 2:**
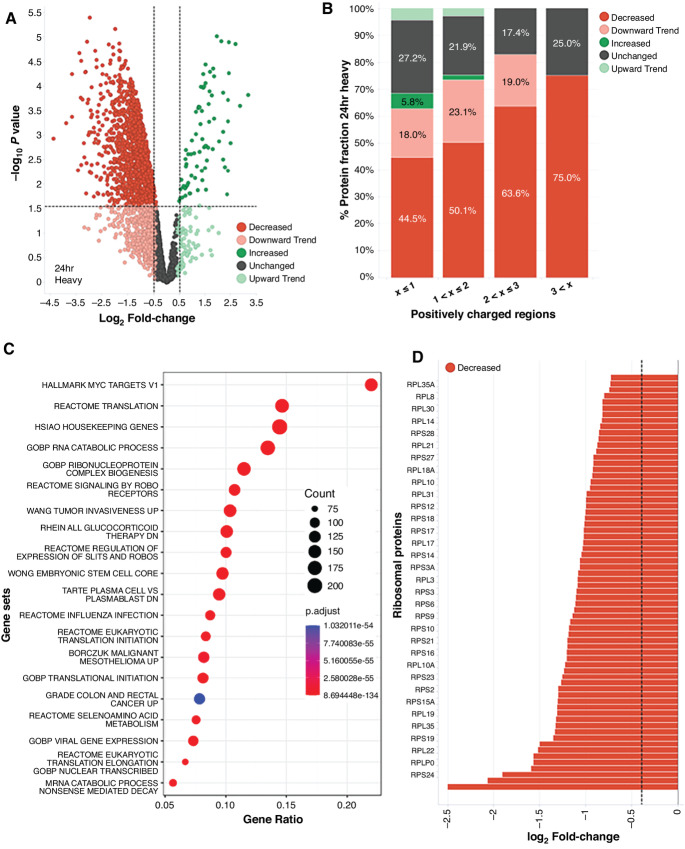
ZKN-157 selectively inhibits new protein synthesis in a sensitive colorectal cancer cell line. **A,** Volcano plot showing heavy-isotope intensities (newly synthesized protein abundance) for 2867 proteins detected at 24-hour timepoint. Protein were classified as follows: Decreased (log_2_FC < −0.4, *P*_adjusted_ < 0.05, red), Downward Trend (log_2_FC < −0.4, *P*_adjusted_ > 0.05, pink), Increased (log_2_FC > 0.4, *P*_adjusted_ < 0.05, dark green), Upward Trend (log_2_FC > 0.4, *P*_adjusted_ > 0.05, light green), Unchanged (−0.4 < log_2_FC < 0.4, *P*_adjusted_ > 0.05, dark gray). Dotted lines indicate −0.5 and 0.5 log_2_FC values. **B,** Bar graph plotting percent protein fraction of heavy-isotope intensity (categorized as in **A**) at 24-hour timepoint versus positively charged regions. Positive charge windows were generated by assigning K and R residues a positive 1 charge and all other amino acid residues a charge of 0. Average charge values were then calculated along a 10-amino acid long sliding window for each protein's amino acid sequence (starting at the first residue and ending at the last complete set of 10 residues). **C,** Overrepresentation analysis and pathway enrichment in significantly downregulated proteins from **A** are shown. Count refers to the number of genes found in each pathway while gene ratio refers to the count divided by all genes in the pathway. **D,** Bar graph showing nascent protein abundance for 68 Ribosomal proteins from heavy-isotope intensity analysis at 24-hour timepoint (categorized as in **A**). The dotted line indicates −0.4 log_2_FC value.

To further characterize the impact of ZKN-157 on protein translation, we conducted a pSILAC proteomics study in SW1417 cells. We treated cells with either ZKN-157 (20 μmol/L) or DMSO and concomitantly exposed them to heavy isotope–labeled forms of arginine and lysine (heavy medium; Fig. 2; Supplementary Fig. S2A). Mass spectrometric quantitation of heavy isotope–labeled and light isotope–labeled peptides was used to assess the impact of ZKN-157 on translation rate and abundance of newly synthesized proteins. At the 6-hour timepoint, no significant change in global abundance of proteins was observed, but the significant decrease in the heavy-to-light isotope ratio (H/L ratio) of 129 out of 1,188 proteins evaluated (Supplementary Fig. S2B) was indicative of a decrease in protein translation rate. Gene Ontology (GO) analysis (16) indicated that a third of these 129 proteins were nucleic acid binding proteins, 60% of which are known to be involved in protein translation including ribosomal proteins, rRNA maturation factors, and translation factors (Supplementary Fig. S2C). At the 24-hour timepoint, we observed a significant decrease in the abundance of newly synthesized molecules for a large subset (1,482/2,867) of the detected proteins (Fig. 2A). Because macrolide antibiotics are known to selectively inhibit protein translation at peptide motifs enriched for positively charged amino acids (9), analysis of the positive charge density of all detected proteins was performed. We observed that, as the density of positively charged regions increased, a larger fraction of proteins was susceptible to translation inhibition (Fig. 2B). Gene overrepresentation analysis (17) indicated that proteins sensitive to ZKN-157–mediated translation inhibition were highly enriched for MYC target genes (Fig. 2C). The ZKN-157–sensitive MYC targets with the highest positive charge density shown in Supplementary Table S1 are enriched for ribosomal proteins, translation factors, and splicing factors. A deeper dive into overrepresentation analysis showed that the top gene sets were highly overlapping with MYC targets, with 16 of the top 20 significant gene sets containing protein translation related proteins including ribosomal proteins, translation initiation, and elongation factors as the key representative genes (Fig. 2C). Because ribosomal proteins have been reported to be highly enriched for positively charged regions (18), it was striking that 68 out of 73 of the detected ribosomal proteins showed a significant decrease in protein synthesis in response to ZKN-157 treatment (Fig. 2D). This impact of ZKN-157 on ribosomal protein synthesis was confirmed by Western blot analysis on a subset of ribosomal proteins (Supplementary Fig. S2D). These data demonstrate that ZKN-157 selectively inhibited the synthesis of a subset of proteins that are components of the cellular protein translation machinery. Specifically, the decrease in translation of almost all ribosomal proteins indicated that ZKN-157 selectively inhibited ribogenesis in these cancer cells.

### ZKN-157–mediated Ribogenesis Inhibition Leads to p21 Induction, Resulting in Cell-cycle Arrest and Apoptosis

**FIGURE 3:**
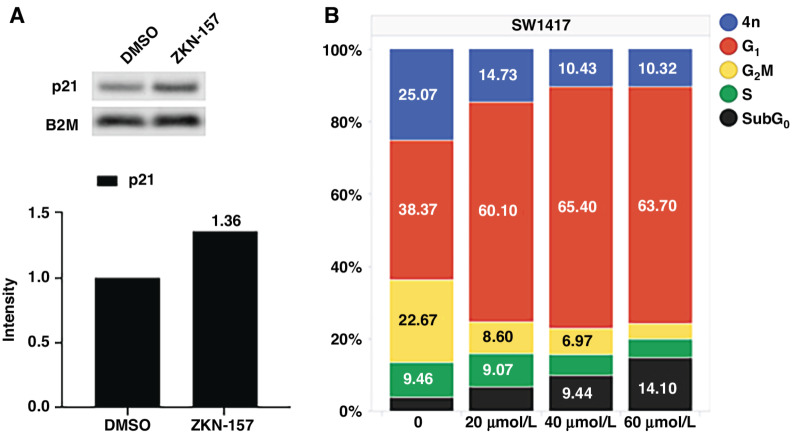
ZKN-157 treatment results in cell-cycle arrest in a sensitive colorectal cancer cell line. **A,** Immunoblots for p21 following 24-hour treatments with DMSO or ZKN-157 (40 μmol/L) are shown. Bar graphs show quantitated intensities normalized to B2M (loading control). **B,** Bar graph plotting percentage of SW1417 cells from cell-cycle phases.

Perturbations that disrupt ribogenesis have been reported to result in cell-cycle arrest or apoptosis (19, 20). To characterize the cellular response to ZKN-157 treatment, we assessed the impact of ZKN-157 on the cell cycle. As seen in Fig. 3A, treatment with ZKN-157 leads to an increase in p21 protein levels. FACS analysis assessing DNA content showed that ZKN-157 treatment triggered a strong G_1_ arrest in SW1417 cells. A significant and dose-dependent increase in the subG_0_ population, indicative of apoptotic cells, was also observed (Fig. 3B). These responses were not observed in the refractory cell line COLO320DM (Supplementary Fig. S3A and S3B). These data demonstrate that ZKN-157 inhibits ribogenesis, leading to induction of p21, and resulting in cell-cycle arrest and apoptosis.

### CMS2 Subtype of Colorectal Cancer is Highly Sensitive to ZKN-157

**FIGURE 4:**
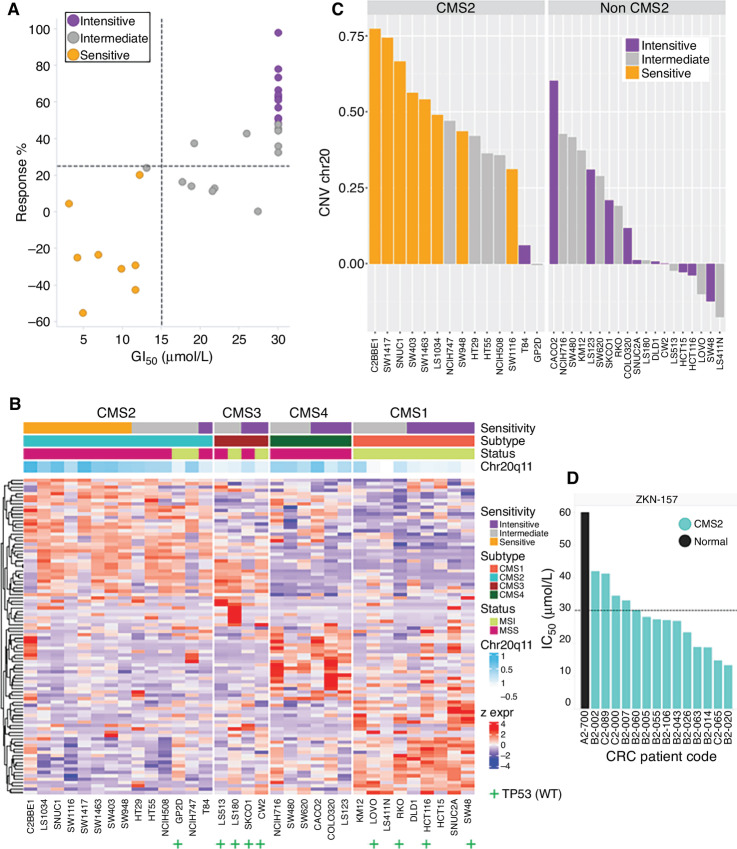
Sensitivity to ZKN-157 enriched in CMS2 subtype and is associated with a distinct molecular signature. **A,** Scatter plot displaying drug sensitivity metrics for ZKN-157 across 33 colorectal cancer cell lines with colors indicating sensitive, intermediate, and insensitive cell lines. Dotted lines indicate response percent (%) = 25 μmol/L (horizontal) and GI_50_ = 15 μmol/L (vertical). **B,** Heat map of gene expression data for CMS subtype genes across colorectal cancer cell lines shows an enrichment of sensitive cell lines in the CMS2 subtype. Row annotations show drug sensitivity, MSI/MSS status, CMS subtypes and average CNV ratios for Chr20q11–13. “+” used to annotate cell lines with WT TP53, unannotated cell lines harbor mutations in TP53. **C,** Bar graph plotting Chr20 CNV ratios for colorectal cancer cell lines, split by CMS2 subtype shows a further enrichment of sensitive cell lines in the CMS2 subtype with Chr20q11-13 CNV gain. **D,** Bar graph plotting IC_50_ values for ZKN-157 across colorectal cancer–derived CMS2 (light blue bars) and normal (black bar) organoids. Dotted line indicates IC_50_ = 30 μmol/L.

To further characterize the response to ZKN-157 we profiled its activity across a panel of 33 colorectal cancer cell lines. We observed that eight cell lines were sensitive to ZKN-157 while 12 cell lines were completely refractory (Fig. 4A). The remaining lines showed an intermediate level of sensitivity to ZKN-157. These colorectal cancer lines have been well characterized in the literature and genomic data associated with them are publicly available. We leveraged these data sources to characterize the clinical and molecular features associated with sensitivity to ZKN-157. Our analysis identified that all eight cell lines sensitive to ZKN-157 belong to the CMS2 subtype of colorectal cancer which is characterized by high MYC and WNT signaling activation, whereas the insensitive and intermediate cell lines distributed across all four CMS subtypes. Another molecular feature shared by all CMS2 cell lines sensitive to ZKN-157 is the microsatellite-stable status (MSS), which is known to harbor significantly more copy-number variations (CNV) than those of the microsatellite-instable status (MSI). Interestingly, our analysis also identified copy-number gains of Chr20q11 as a novel genomic feature present in all CMS2 cell lines sensitive to ZKN-157 (Fig. 4B and C). To further validate the sensitivity associated with the CMS2 subtype, we evaluated ZKN-157 across a panel of CMS2, patient-derived colorectal cancer organoids. ZKN-157 inhibited proliferation across the CMS2, patient-derived organoids without affecting growth of a model organoid derived from normal colon (Fig. 4D). These data demonstrate that ZKN-157 targets colon cancer cells of the CMS2 subtype characterized by high MYC and WNT pathway activity.

### DNA-intercalating Agents Demonstrate Robust Combination Synergy with ZKN-157

**FIGURE 5:**
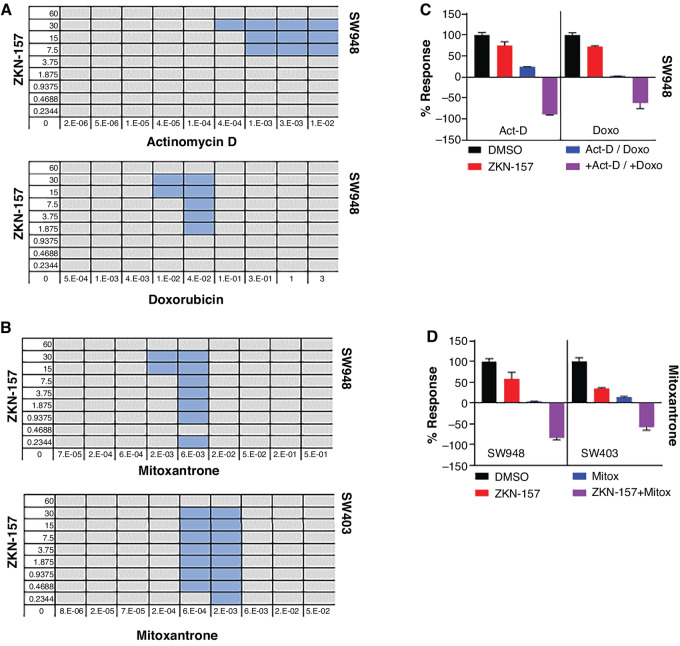
ZKN-157 demonstrates synergy with DNA-intercalating agents in CMS2 cell lines. **A,** Matrices showing BI scores for combinations of ZKN-157 with actinomycin D and doxorubicin on SW948 cells. Dose combinations demonstrating synergy are highlighted in blue. **B,** Matrices showing BI scores for combinations of ZKN-157 with mitoxantrone on two cell lines: SW948 and SW403. Single-agent concentrations (in μmol/L) in all first columns and bottom rows for all matrices. **C,** Percent response bar graph showing ZKN-157′s efficacy enhanced in combination with actinomycin D (Act-D) and doxorubicin (Doxo) in SW948 cells. Bars are color coded as follows: DMSO (black), ZKN-157 (30 μmol/L, red), Act-D (10 nmol/L, blue), Doxo (330 nmol/L, blue), ZKN-157+Act-D and ZKN-157+Doxo (purple). **D,** Percent response bar graph showing ZKN-157’s efficacy enhanced in combination with mitoxantrone (Mitox) in SW948 and SW403 cells. Bars are color coded as follows: DMSO (black), ZKN-157 (30 μmol/L, red), Mitox (6 nmol/L, blue; SW948), Mitox (5.56 nmol/L, blue; SW403), and ZKN-157+Mitox (purple).

Development of novel cancer therapies depends not only on single-agent activity, but also requires combinations with existing agents to achieve maximal clinical efficacy. While additivity between two cancer agents can be sufficient, synergy is highly desired to maximize the anticancer effect. To identify agents that could synergize with ZKN-157, we screened a panel of 14 approved drugs that are standard-of-care chemotherapeutics or targeted therapies used to treat colorectal cancer. The potential for combination synergy was evaluated in SW403 (a CMS2 line) using the BI model (21). A majority of the drugs tested were classified as having an additive effect in combination with ZKN-157 (Table 1), but interestingly, the three DNA-intercalating agents consistently showed synergistic effects in combination with ZKN-157 in two CMS2 colorectal cancer cell lines (Fig. 5). In the two CMS2 lines, cytostatic concentrations of single agents, when combined, generated a robust cytotoxic response, demonstrating the potential synergy of DNA-intercalating agents with ZKN-157 (Fig. 5C and D).

**TABLE 1:**
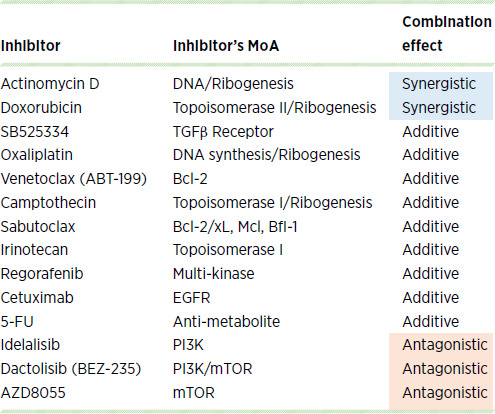
Table showing 14 inhibitors tested in combination with ZKN-157. Combination effects were color coded blue (synergism) or orange (antagonism) according to the scores obtained from the BI analysis

## Discussion

Ribosome production in a rapidly dividing cell requires immense cellular resources (22), and thus targeting ribosomes in cancer represents an attractive approach to treatment. As with targeting universal cellular processes to treat cancer through chemotherapy or antimetabolite therapy where the therapeutic window becomes limiting, targeting catalytic ribosome steps suffers from the same drawback. We have taken an orthogonal approach by leveraging rapid advances in chemistry coupled with a detailed understanding of ribosome biology. First, we have used our advanced chemistry platform to create a library of novel macrolides (11) which occupy a new chemical space with increased potential to target the human ribosome due to the high degree of conservation of the macrolide binding site across species (10). Macrolides bind to the nascent peptide exit tunnel, and thus act as allosteric modulators of the ribosome, overcoming issues with inhibiting the highly conserved catalytic steps involved in protein synthesis. Second, we have identified a subset of molecules, including ZKN-157, which exploit cancer ribosome heterogeneity to selectively target a subset of colorectal cancer cell lines.

ZKN-157 demonstrates two levels of selectivity. First, protein translation inhibition occurs preferentially in some cell types and not others, exemplified by SW1417 versus COLO320DM. Although previously hypothesized (23), this more selective ribosome inhibition in one cell line versus another is the first demonstration of differential ribosome targeting. A second level is the propensity of ZKN-157 to selectively inhibit translation of proteins with a high density of positively charged regions. This activity was less surprising given that the mechanism of protein translation inhibition by macrolide antibiotics is well described, and the process of ribosome-mediated protein synthesis is conserved across prokaryotes and eukaryotes. ZKN-157 and macrolide antibiotics show similar patterns of selective translation inhibition of proteins that contain regions with high density of positively charged amino acids, in CMS2 colon cancer cells and bacterial cells, respectively. These data suggest that similar to the activity of macrolide antibiotics in bacteria, ZKN-157 acts as selective allosteric ribosome inhibitor in the sensitive cancer cell lines. It is hypothesized that the positively charged amino acids interact with the negatively charged rRNA lining the NPET which slows down the passage of the nascent peptide through the NPET (18). We observed that as the density of positively charged regions increased, a larger fraction of proteins was sensitive to translation inhibition by ZKN-157 (Fig. 2B). The slow translation of sequences enriched for positively charged amino acids is likely further exacerbated by the presence of ZKN-157 in the NPET, resulting in ribosome stalling and translation inhibition. Published reports indicated that nucleic acid binding proteins and ribosomal proteins in particular are highly enriched for positively charged regions (18). Additional classes of proteins enriched for positively charged regions include chromosomal and nucleolar proteins as well as proteins localized to the mitochondria. Consistent with these reports, all these classes of proteins are enriched among the proteins impacted by ZKN-157. This finding is novel as all known human ribosome inhibitors target catalytic steps and do not show selectivity for specific protein sequences.

ZKN-157 also sheds new light on mechanisms used by cancer to co-opt normal cellular processes for disease progression. It was unexpected that ZKN-157–induced protein translation inhibition significantly impacted proteins that map to the MYC pathway. Hence it was not surprising that cell line profiling across 33 colorectal cancer lines identified eight that were sensitive to ZKN-157 belong to the CMS2 subtype of colorectal cancer (12). The CMS2 subtype accounts for 37% of colorectal cancer and is characterized by an activated WNT pathway and high MYC signaling (12). Sensitivity in theCMS2 subtype was confirmed in a panel of CMS2 patient-derived organoids, highlighting the potential for clinical translation of this novel mechanism to patients that harbor this subtype of colorectal cancer (7). The spectrum of MYC targets whose protein synthesis was affected were predominantly those that contain high density of positively charged regions and are involved in protein translation either directly, that is, eukaryotic initiation factors, or indirectly, that is, ribosomal proteins that are components of ribosomes.

The nucleolus, the site of ribogenesis, plays a critical role in regulating cell-cycle progression, senescence, and apoptosis (20). Perturbations that interfere with ribogenesis trigger a nucleolar stress response that leads to cell-cycle arrest and apoptosis via p53-dependent and p53-independent mechanisms (24). Consistent with this, inhibition of ribogenesis by ZKN-157 led to p21 induction, resulting in robust G_1_ arrest and induction of apoptosis. The colorectal cancer cell line profiling data indicate that sensitivity to ZKN-157 does not require wild-type (WT) p53 (Fig. 4B).

Cancer therapy is rarely based on single agents. However, most combinations used in cancer therapy show additivity. We observed that combining ZKN-157 with clinically used DNA-intercalating agents, that are known to inhibit rRNA expression (14), resulted in a robust cytotoxic response in two CMS2 lines. Among the different types of therapeutics tested, only DNA-intercalating agents demonstrated synergy with ZKN-157. These data highlight the potential to design mechanism-based combinations that target vulnerability of the CMS2 subtype of colorectal cancer to ribogenesis inhibition and thereby provide opportunities to optimize therapy with ZKN-157. CMS2 tumors often arise in the distal colon, tend to be WT for KRAS and appear to be more sensitive to cetuximab (25). Our results suggest that selectively targeting ribosome could be an additional therapeutic approach for refractory patients that can be tested in clinical studies.

Many small-molecule anticancer agents, especially kinase inhibitors, demonstrate the ability to inhibit targets at a cellular level at submicromolar concentrations. However, ZKN-157 requires higher micromolar concentrations to produce an effect on protein synthesis. An assessment of target protein abundance showed that while kinases are present at approximately 10^3^ copy numbers of protein per cell, ribosomes have 10^6^–10^7^ copy numbers per cell (26), and it is likely higher in cancer cells. On the basis of average cytoplasmic volume of 1 picoliter (27), the estimated ribosomal concentration ranges from 1 to 10 μmol/L, which is approximately 1,000-fold greater than the concentration of typical kinase drug targets. The potency of ZKN-157 in sensitive lines is consistent with this high concentration of ribosomes in cells. Fortuitously, RMAs retain the excellent drug-like properties and high tissue exposure that is inherent to the macrolide class of antibiotics (28). Macrolides have demonstrated high tissue distribution and long-terminal half-life in preclinical species as well as in humans (29, 30). In single-dose pharmacokinetic studies, azithromycin demonstrated tissue to plasma ratio > 100 across multiple tissues in both rat and dog. In repeat dose pharmacokinetic studies, azithromycin showed 6- to10-fold accumulation in various tissues in both species (31). It remains to be seen whether ZKN-157 retains the properties of long half-life and high tissue exposure of macrolides, thereby enabling coverage of high abundance ribosomes.

The activity and selectivity of ZKN-157 exploits two factors: (i) addiction of CMS2 subtype to high protein translation capacity; and (ii) ribosome heterogeneity, that enables selective targeting of ribosomes in this subtype of cancers. Mutations and copy-number alterations in RPGs, dysregulated expression of snoRNAs that regulate chemical modification of specific rRNA residues, and dysregulation of various steps involved in ribogenesis prevalent in sensitive CMS2 subtype likely enable ZKN-157 to selectively target ribosomes in this subtype of colorectal cancer. Additional studies will be needed to further characterize the specific binding interactions resulting from ribosome alterations associated with sensitivity to ZKN-157.

We have demonstrated a novel mechanism of action for ZKN-157 providing evidence that ribosome heterogeneity in cancer can be exploited by this novel class of ribosome inhibitors to develop selective ribogenesis inhibitors. This work has translational implications for CMS2 colorectal cancers and potentially other cancer subtypes that harbor high MYC activation and thus potential addiction to high protein translation capacity.

## Supplementary Material

Figure S1 Imaging assay demonstrating that ZKN-157 inhibits translation of newly synthesized proteins

Figure S2 Results for pSILAC Mass Spec study demonstrating ZKN-157 inhibits translation of a subset of proteins

Figure S3 FACS analysis demonstrating ZKN-157 selectively induces cell cycle arrest and apoptosis

Supplementary Table 1 Table showing the top 20 MYC gene targets with the highest positive charge density

Supplementary Data 1 ZKN-157- chemical characterization

Supplementary Methods 1 Supplementary methods
