# Supplementary material for: A Novel Class of Ribosome Modulating Agents Exploits Cancer Ribosome Heterogeneity to Selectively Target the CMS2 Subtype of Colorectal Cancer
Source: Cancer Res Commun. 2023 Jun 5;3(6):969–79. doi: 10.1158/2767-9764.CRC-22-0469 (PMC10241187; doi:10.1158/2767-9764.CRC-22-0469)
Supplement: Figure S1 — Imaging assay demonstrating that ZKN-157 inhibits translation of newly synthesized proteins [file crc-22-0469-s01.docx]

**
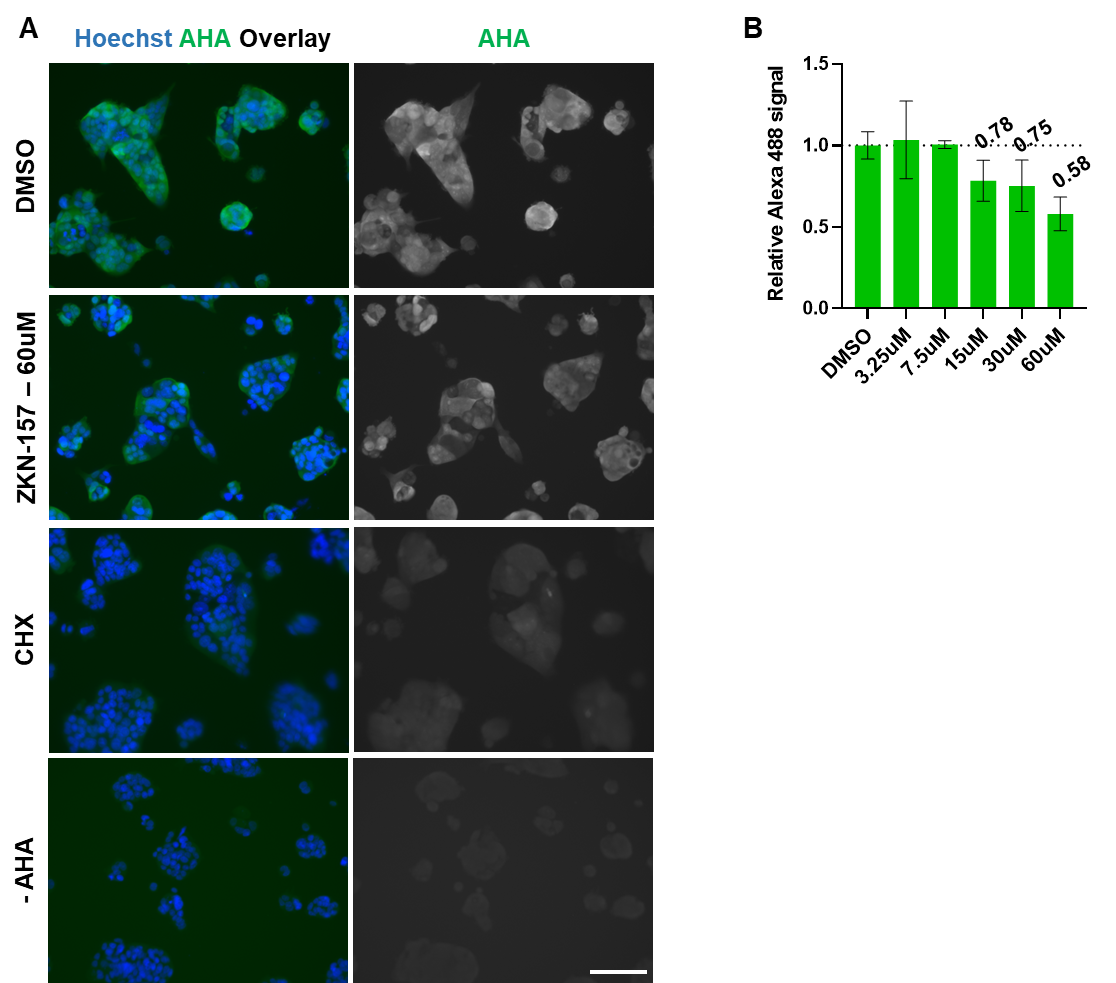
**

**Supplementary Figure S1**

ZKN-157 inhibits translation of newly synthesized proteins. **A.** Panels showing representative immunofluorescence images of SW1417 cells stained for DNA (Hoechst, blue) and AHA incorporated into newly synthesized proteins (Alexa 488, green) after 24hr treatment. -AHA (no AHA reagent added to medium); Cycloheximide (CHX) (positive control for translation inhibition); DMSO (negative control); and ZKN-157 treatment (60uM). Sale bar = 100um. **B,** Bar graph from representative experiment plotting relative Alexa 488 signal from SW1417 cells treated with DMSO and 5 doses of ZKN-157 for 24hr. Error bars are standard deviations.
