# Supplementary material for: A Novel Class of Ribosome Modulating Agents Exploits Cancer Ribosome Heterogeneity to Selectively Target the CMS2 Subtype of Colorectal Cancer
Source: Cancer Res Commun. 2023 Jun 5;3(6):969–79. doi: 10.1158/2767-9764.CRC-22-0469 (PMC10241187; doi:10.1158/2767-9764.CRC-22-0469)
Supplement: Figure S2 — Results for pSILAC Mass Spec study demonstrating ZKN-157 inhibits translation of a subset of proteins [file crc-22-0469-s02.docx]

**
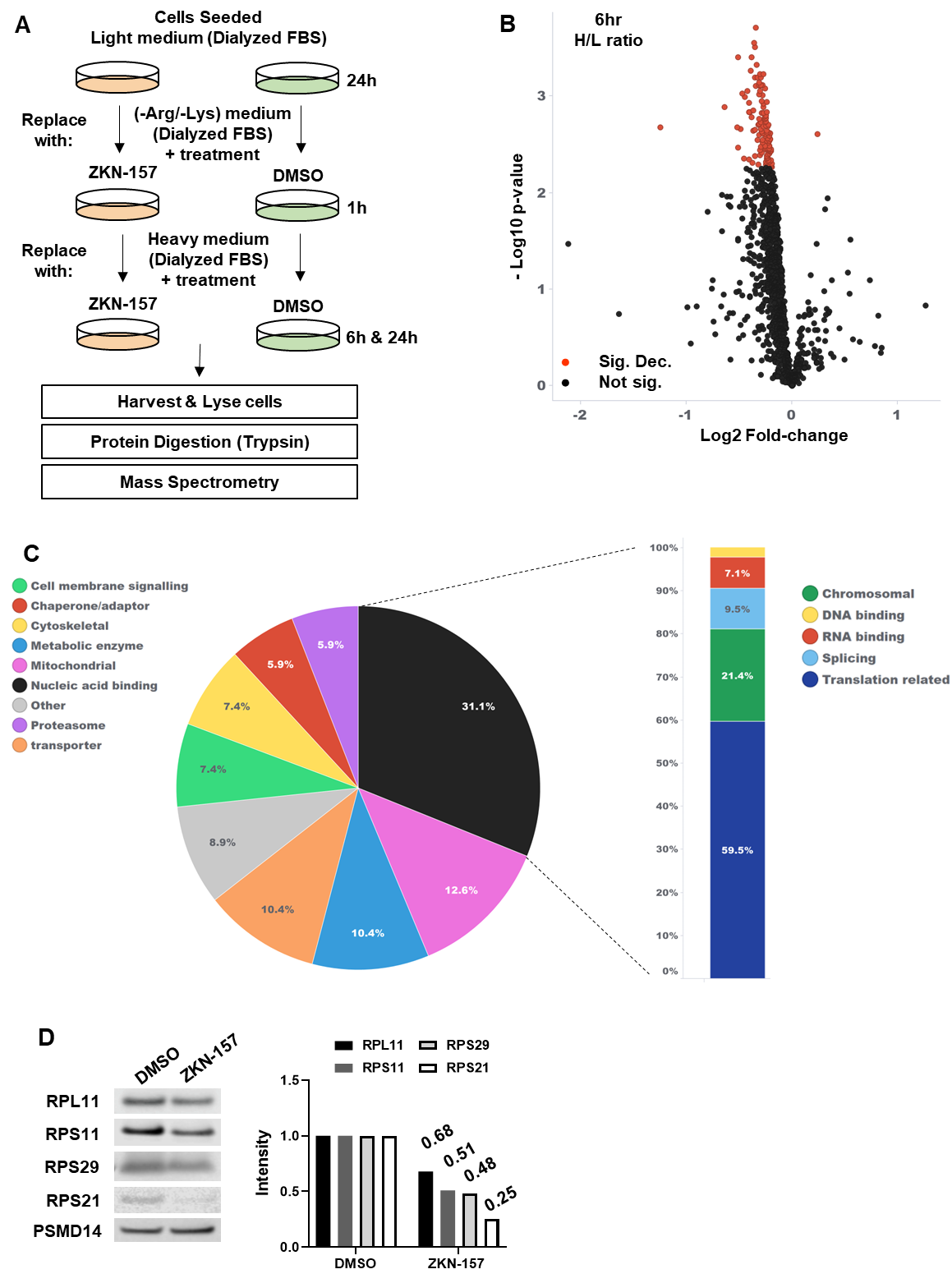
**

**Supplementary Figure S2**

ZKN-157 inhibits translation of a subset of proteins. **A,** Cartoon showing pSILAC assay’s workflow. **B,** Volcano plot showing heavy-to-light (H/L) ratios for 1188 proteins detected at 6-hour time point. Proteins displaying significantly decreased (adjusted p-value < 0.05) H/L ratios (129) are depicted in red circles. Detected proteins not significantly affected by ZKN-157 are depicted in black circles. **C,** Pie chart showing 129 significantly decreased detected proteins grouped in classes curated from Gene Ontology (GO) classification. Bar graph shows 5 subclasses within the Nucleic acid binding class. **D,** Immunoblots for RPL11, RPS11, RPS29, and RPS21 showing samples from SW1417 cells treated with DMSO or ZKN-157 (20uM) for 72 hours. Bar graph showing quantitated intensities normalized to PSMD14 (loading control).
