# Supplementary material for: A Novel Class of Ribosome Modulating Agents Exploits Cancer Ribosome Heterogeneity to Selectively Target the CMS2 Subtype of Colorectal Cancer
Source: Cancer Res Commun. 2023 Jun 5;3(6):969–79. doi: 10.1158/2767-9764.CRC-22-0469 (PMC10241187; doi:10.1158/2767-9764.CRC-22-0469)
Supplement: Figure S3 — FACS analysis demonstrating ZKN-157 selectively induces cell cycle arrest and apoptosis [file crc-22-0469-s03.docx]

**
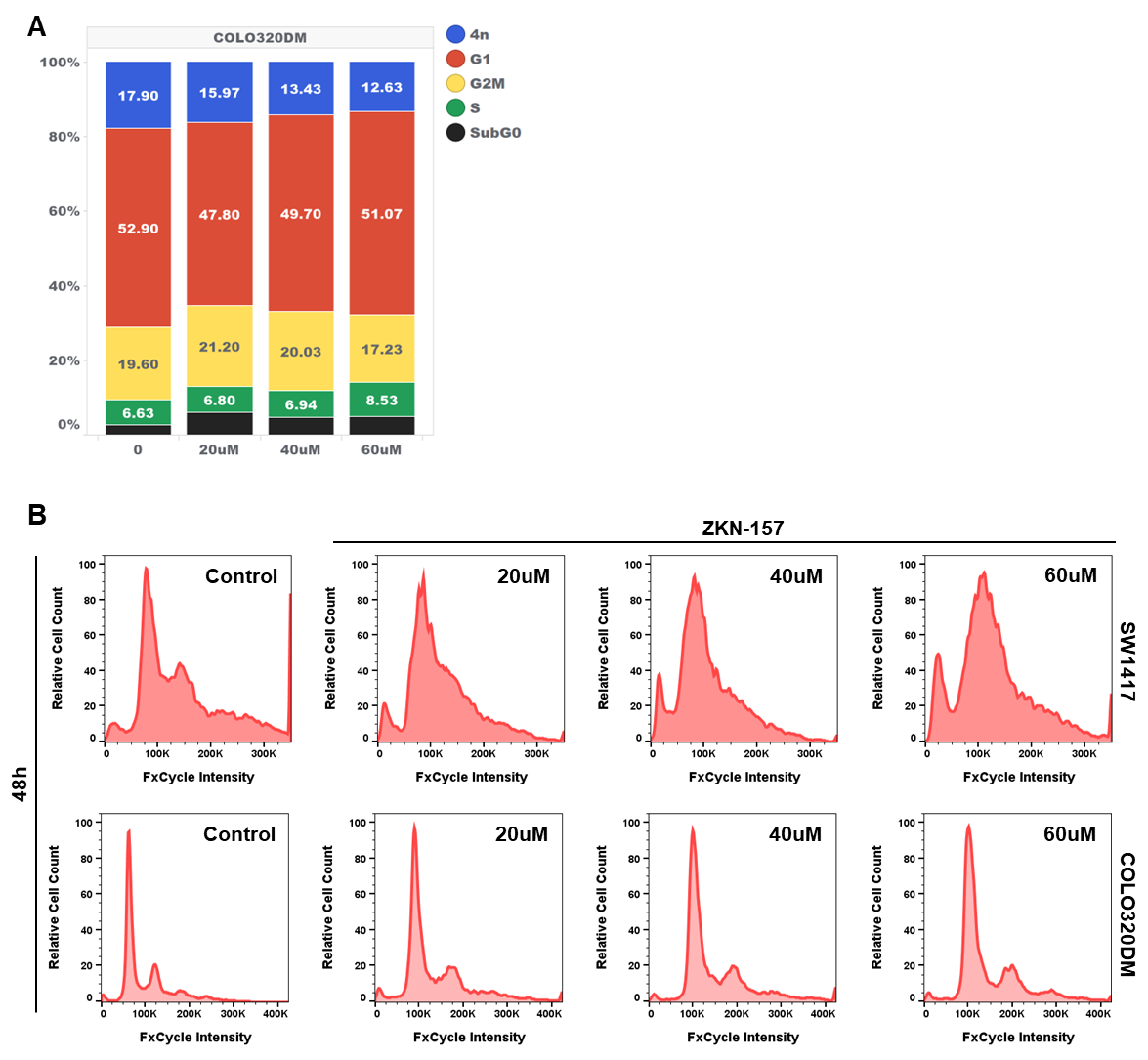
**

**Supplementary Figure S3**

ZKN-157 selectively induces cell cycle arrest and apoptosis. **A,** Bar graph plotting percentage of COLO320DM cells from cell cycle phases: **B,** Graphs showing DNA content analysis by flow cytometry for control (0uM) and 48-hour ZKN-157 treatment (20, 40, and 60uM) conditions from SW1417 and COLO320DM cell lines.
