# Supplementary material for: A Novel Class of Ribosome Modulating Agents Exploits Cancer Ribosome Heterogeneity to Selectively Target the CMS2 Subtype of Colorectal Cancer
Source: Cancer Res Commun. 2023 Jun 5;3(6):969–79. doi: 10.1158/2767-9764.CRC-22-0469 (PMC10241187; doi:10.1158/2767-9764.CRC-22-0469)
Supplement: Supplementary Table 1 — Table showing the top 20 MYC gene targets with the highest positive charge density [file crc-22-0469-s04.docx]

| **Protein** | **Average window charge** | **% windows above threshold** |
| --- | --- | --- |
| **RPL34** | 2.73 | 88 |
| **SRSF2** | 3.04 | 83 |
| **RPS6** | 2.75 | 78 |
| **SRSF7** | 2.76 | 76 |
| **RPL18** | 2.62 | 76 |
| **RPL6** | 2.61 | 76 |
| **RPL14** | 2.29 | 70 |
| **NOP16** | 2.32 | 70 |
| **SRSF1** | 2.05 | 63 |
| **SSB** | 1.95 | 63 |
| **RPS10** | 1.87 | 59 |
| **EIF4H** | 1.69 | 59 |
| **RPL22** | 2.03 | 59 |
| **SNRPD2** | 2.05 | 57 |
| **SERBP1** | 1.88 | 56 |
| **CBX3** | 1.87 | 55 |
| **SNRPD1** | 2.03 | 51 |
| **EIF2S2** | 1.87 | 50 |
| **EIF1AX** | 1.81 | 49 |
| **EIF3J** | 1.85 | 48 |
| Baseline***** | 1.20 | 34 |

**Supplementary Table 1.**

Table showing the top 20 MYC gene targets with the highest positive charge density. Baseline (*) represents the median value across all proteins detected by Mass Spectrometry. Methods used to calculate positive charge metrics are detailed in the Supplementary Methods section.
