# Supplementary material for: A Novel Class of Ribosome Modulating Agents Exploits Cancer Ribosome Heterogeneity to Selectively Target the CMS2 Subtype of Colorectal Cancer
Source: Cancer Res Commun. 2023 Jun 5;3(6):969–79. doi: 10.1158/2767-9764.CRC-22-0469 (PMC10241187; doi:10.1158/2767-9764.CRC-22-0469)
Supplement: Supplementary Data 1 — ZKN-157- chemical characterization [file crc-22-0469-s05.docx]

0.992

O

7.5

1.928

7.0

0.972

N

6.5

7.263

7.244


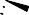

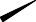

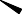


CO2H

CO2H

OH

HO2C

N

O

N

O

O

8.5

8.0

7.224

HO

6.933

6.927

6.906

6.863

6.858

6.843

6.0

0.954

5.0

4.5

1.044

0.941

4.0

3.039

1.169

1.941

3.5

1.103

0.906

0.841

1.032

3.0

5.080

2.796

11.310

2.5

3.940

1.171

2.0

1.098

1.085

1.949

1.5

17.255

3.000

1.0

Chemical Shift (ppm)

4.581

4.547

5.5

4.468

4.450

4.212

4.187

3.789

3.615

3.452

3.426

3.407

3.310

3.278

3.036

3.001

2.872

2.782

2.761

2.745

2.709

2.671

1.991

1.917

1.813

1.780

1.493

1.396

1.372

1.365

1.355

1.315

1.300

1.071

1.055

**Compound ID: ZKN-157 citric acid salt**

**EC4771-5-P1N1 MeOD Bruker_CD-E_400MHz**

O

O

O

9.5

9.0

**LCMS Report**

===================================

Compound ID : ZKN-157 citric acid salt Sample ID : EC4771-5-P1A1

Injection Vol : 3ul Location : vial98

Acq Method : D:\method\0-60AB_1min.lcm

Org DataFile : D:\DATA\2021\2110\211012\EC4771-5-P1A1.lcd

Injection Date : 2021/10/12 15:16:08 Instrument : CAS-CD-LCMS-AB

mAU

Chromatogram

PDA Multi 1 220nm,4nm

500

0.448

0

0.438

0.0 0.1 0.2 0.3 0.4 0.5 0.6 0.7 0.8 0.9 1.0

min

50

mAU

PDA Multi 2 254nm,4nm

0.448

0

0.437

0.0 0.1 0.2 0.3 0.4 0.5 0.6 0.7 0.8 0.9 1.0

min

MS Chromatogram

(x10,000,000)

17,217,512

(-0.017min Delay)

1.00

0.00

-0.0 0.1 0.2 0.3 0.4 0.5 0.6 0.7 0.8 0.9

min

TIC(+)@1

**===========================================================**

**Integration Result**

**===========================================================**

PDA Ch1 220nm

| Peak# Ret. Time Height Height% USP Width Area Area% | | | | | | |
| --- | --- | --- | --- | --- | --- | --- |
| 1 | 0.438 | 3216 | 0.375 | 0.021 | 1353 | 0.153 |
| 2 | 0.448 | 854484 | 99.625 | 0.023 | 885786 | 99.847 |
| PDA Ch2 254nm |  |  |  |  |  |  |
| Peak# Ret. Time Height Height% USP Width Area Area% | | | | | | |
| 1 | 0.437 | 1992 | 3.486 | 0.015 | 897 | 1.029 |
| 2 | 0.448 | 55166 | 96.514 | 0.044 | 86284 | 98.971 |

Mass Spectrum

RetTime: 0.448 DateFile: D:\DATA\2021\2110\211012\EC4771-5-P1A1.lcd

8000000

345.8

7000000

690.5

6000000

5000000

4000000

3000000

2000000

1000000

100 200 300 400 500 600 700 800 900 1000


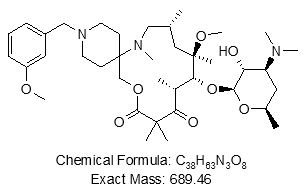
m/z

HPLC REPORT

Compound ID : ZKN-157 citric acid salt Sample ID : EC4771-5-P1AS1

Injection Date : Tue, 12. Oct. 2021 17:18:39 Location : P2-A-03

Inj. Vol. : 6.0 uL

Data Filename : D:\DATA\NON-GXP\2021\10\20211012_02\EC4771-5-P1AS1.D

Instrument : CAS-CD-LCMS-G

Acq Method : D:\data\non-GXP\2021\10\20211012_02\0-60AB_20MIN_ Poroshell_15CM.M

| DAD1 E, Sig=220,4 Ref=off (D:\DATA\NON-GXP\2021\10\20211012_02\EC4771-5-P1AS1.D) | | | | |
| --- | --- | --- | --- | --- |
| mAU | 4.433  7.997 8.177  8.891 | | | |
| 500 |  |  |  |  |
| 400 |  |  |  |  |
| 300 |  |  |  |  |
| 200 |  |  |  |  |
| 100 |  |  |  |  |
| 0 |  |  |  |  |
|  | 5 | 10 | 15 | min |

Report

==================================================================

| Signal ->: DAD1 # Meas. Ret. | | E, Sig=220,4 Ref=off Height Width | | Area | Area % |
| --- | --- | --- | --- | --- | --- |
| 1 | 4.433 | 1.937 | 0.035 | 4.236 | 0.057 |
| 2 | 7.702 | 5.102 | 0.041 | 13.933 | 0.188 |
| 3 | 7.831 | 5.386 | 0.042 | 15.153 | 0.205 |
| 4 | 7.997 | 1.564 | 0.048 | 5.185 | 0.070 |
| 5 | 8.177 | 590.525 | 0.179 | 7255.604 | 97.959 |
| 6 | 8.735 | 23.763 | 0.061 | 95.724 | 1.292 |
| 7 | 8.891 | 3.037 | 0.080 | 16.905 | 0.228 |

Page 1 of 1
