# Supplementary material for: A Novel Class of Ribosome Modulating Agents Exploits Cancer Ribosome Heterogeneity to Selectively Target the CMS2 Subtype of Colorectal Cancer
Source: Cancer Res Commun. 2023 Jun 5;3(6):969–79. doi: 10.1158/2767-9764.CRC-22-0469 (PMC10241187; doi:10.1158/2767-9764.CRC-22-0469)
Supplement: Supplementary Methods 1 — Supplementary methods [file crc-22-0469-s06.docx]

**Supplementary Materials and Methods**

**Metabolic labeling**

Cells were washed once with warm -Met media and incubated in -Met media with DMSO, 40uM ZKN-157, or 50ug/mL CHX for 30 minutes. 50uM of AHA (L- Azidohomoalanine) reagent was added to the -Met media for each condition and incubated for 1 hour. Cells for each condition were washed once with complete media (culturing media) and incubated for 1 hour in complete media. Cells were washed with PBS and 200uL lysis buffer containing 50mM Tris HCl at pH 8.0 (Thermo), 1%SDS (Fisher Chemicals), 1:100 of 100X protease and phosphatase inhibitor (Pierce Biotech) and 1:1000 of 20X Universal Nuclease for cell lysis (Invitrogen) were added per well to the plate for SW1417. For COLO320DM, the cells were collected in a 15mL tube and centrifuged at 1200rpm for 5 minutes, washed with PBS once and centrifuged again; 200uL of lysis buffer were added to each tube. For SW1417, the cells were scraped and transferred to 1.5mL tube. The samples were kept on ice for 15 minutes, followed by vortexing for 5 minutes and spinning at 15000g for 10 minutes at 4C. The supernatant was transferred into a new 1.5mL tube. Protein concentration was determined using BCA assay (Pierce Biotech). 200ug of protein from all cell lysates were prepared in 50uL and then brought up to final volume (60uL) with deionized water. For click chemistry, the Click-iT Protein Reaction Buffer Kit (Invitrogen) was used. All reagents in the kit were reconstituted and click reaction was completed following manufacturer's instructions. The dried samples were reconstituted in 25uL of 1X LDS sample loading buffer (Invitrogen) and resolved by SDS-PAGE. The Gel was transferred and blocked for 1h in 1:20000 of IRDye® 800CW Streptavidin (LiCor). The membrane was washed 3 times with 1X TBST (Thermo) for 5 minutes.

**In-cell Metabolic labeling**

Cells were seeded at a density of 3x10^4^ cells in a 96-well black clear-bottom plates (Sigma) in triplicate for 48 hours. Cells were treated with DMSO or ZKN-157 at various concentrations for 24 hours, and 50ug/mL of CHX (Sigma) for 2 hours prior to incubation in methionine free (-Met) media. Cells were washed once with warm -Met media and incubated in -Met media with treatments for 30 minutes. 50uM of AHA (L- Azidohomoalanine) reagent was added to the -Met media for each condition and incubated for 3 hours. Cells were washed with PBS and fixed using 3.7% formaldehyde in PBS followed by permeabilization using 0.5% Triton X-100. The click reaction and detection were performed using the Click-iT™ AHA Alexa Fluor™ 488 Protein Synthesis HCS Assay kit (Invitrogen). Plates were read on Biotek Cytation 5 plate-reader (Agilent) and data was analyzed using GraphPad Prism software. Images were acquired on the EVOS™ M5000 Imaging System (Invitrogen) with 20X objective lenses.

**Mass Spectrometry**

LC-MS/MS Analysis: Dried peptides were resuspended in 0.1% formic acid and then analysed by online nanoflow LC‐MS/MS using an Orbitrap Exploris 240 mass spectrometer (Thermo Scientific) coupled to an Ultimate 3000 RSLCnano (Thermo Scientific). Peptides (equivalent to 2µg protein) were injected on an Acclaim PepMap 100 C18 LC trap column (100 μm ID ×20 mm, 5 μm, 100 Å) followed by separation on an EASY-Spray nanoLC C18 column (75 μm ID ×500 mm, 2 μm, 100 Å) at a flow rate of 250nl/min. Buffer A contained 0.1% formic acid in water and Buffer B contained 0.1% formic acid in acetonitrile. Peptides were separated with a linear gradient of 2–37% Buffer B over 213 min followed by a step from 37 to 80% Buffer B in 1 min; then, the column was washed with 80% Buffer B and re-equilibrated to 2% Buffer B to complete the 240 min run. The Orbitrap Exploris 240 was operated in positive-ion data-dependent mode. Precursor ion (MS1) scans were performed in the Orbitrap mass analyzer in the range of 350–1,400 m/z, with a resolution of 120,000 (at 200 m/z). Precursor ions were isolated using a quadrupole mass filter with an isolation width of 1.6 m/z and fragmented using higher energy collision dissociation (HCD) with a collision energy of 30%. MS/MS fragment ions were analysed in the Orbitrap mass analyser with a resolution of 15,000 (at 200 m/z). The database search was performed using the *Homo sapiens* reference proteome from UniProtKB. Oxidation of methionine and acetylation of protein N-terminus were allowed as variable modifications, while carbamidomethylation of cysteine was allowed as a fixed modification. The estimated false discovery rate was set to 1% at the peptide and protein levels. A maximum of two missed cleavages were allowed. Reverse hits, contaminants, and proteins only identified by site were removed before further analysis. Protein groups were further filtered to contain at least two unique peptides. Hypothesis testing was performed in R (version 4.1.0) using the Limma package available in the R/Bioconductor repository and p-values were corrected for multiple hypothesis testing using the Benjamini-Hochberg method. Proteins with a corrected p-value <0.05 were considered significantly differential.

**Immunoblotting**

Cells were plated at a density of 0.75x10^6^ cells/60mm dish. After 48 hours, cells were treated with either DMSO or ZKN-157 at specified concentrations for specified time periods. Following treatment, cells were lysed with 250mL of RIPA buffer (RIPA lysis and Extraction Buffer, Thermo Scientific) supplemented with 1X protease and phosphatase inhibitors (Halt™ Protease and Phosphatase Inhibitor Cocktail (100X), Thermo Scientific) and nuclease (1:1000, Universal Nuclease, Thermo Scientific). Proteins were separated on 4-12% gradient gels (NuPAGE Bis-Tris protein gels, Invitrogen) using SDS running buffer (NuPAGE MES SDS Running Buffer, Invitrogen). Primary antibodies: RPL11 (Cell Signaling Technology, Cat# 18163, RRID:AB_2798794, 1:1000), RPS11 (Abcam, ab175213, 1:1000), RPS21 (Thermo Fisher Scientific Cat# PA5-51914, RRID:AB_2646784, 1:1000), RPS29 (Thermo Fisher Scientific Cat# PA5-41744, RRID:AB_2608377, 1:1000), Fibrillarin (Cell Signaling Technology Cat# 2639, RRID:AB_2278087, 1:1000), p21 Waf1/Cip1 (Cell Signaling Technology Cat# 2947, RRID:AB_823586, 1:1000), β2-microglobulin (Cell Signaling Technology Cat# 12851, RRID:AB_2716551, 1:5000), and PSMD14 (Cell Signaling Technology Cat# 4197, RRID:AB_11178935, 1:5000).

**Organoid Culture**

Organoid culture components are listed in table below. For cell viability screening assay, organoids were passaged and seeded at high density. After one day of expansion, organoids were harvested by adding Dispase at a final concentration of 1 mg/mL to the culture medium and incubating at 37°C to digest Matrigel (MG)/Basement Membrane Extract (BME). Then, organoids were collected and size-selected between 40 and 100μm using cell strainers. 250 organoids were dispensed/well in a total volume of 40μL of organoid culture medium (with 5% extracellular matrix) in triplicates for all test conditions in ultra-low attachment (ULA) 384-well plates. Two plates were simultaneously seeded from the same organoid suspension, one for CellTiter-Glo measurement directly after plating (“Day 0”) and one to be measured 7 days after incubation with 9 doses in 3-fold dilutions of ZKN-157 with 60uM as top concentration with normalization to 0.3% vehicle in all test wells. Immediately after plating, all compounds were dispensed on the “Day 7” assay plate using the Tecan D300. Staurosporine was used at 2μM as a positive control for cell death in all “Day 7” assay plates. After 7 days of exposure to ZKN-157, luminescence was measured using Cell Titer-Glo 3D on a Tecan Spark 10M plate reader. IC50 values were calculated using the GRmetrics package.

| **Reagent** | **CSM** | **CTM** |
| --- | --- | --- |
| N-Ac | √ | √ |
| A83-01 | √ | √ |
| B27 Supplement | √ | √ |
| EGF | √ | √ |
| Gastrin | √ | √ |
| Noggin | √ | √ |
| Nicotinamide | √ | √ |
| Primocin | √ | √ |
| R-spo3 | √ | √ |
| SB202190 (P38i) | √ | √ |
| NGS-Wnt | √ | * |
| Matrigel | √ | * |
| BME | * | √ |

**Bioinformatic analysis**

Amino Acid Residue Charge Analysis: Protein amino acid sequences were downloaded from Uniprot (Homo sapiens & reviewed_yes) and were cross referenced with mass spectrometry data using the uniport protein ID. Proteins from differential expression analysis with adjusted p value <0.05 were analyzed for amino acid sequence charge distributions and gene set enrichment. To investigate consecutive positively charged amino acid sequences effect on ribosomal translation, we focused on positively charged amino acids only, assigning lysine (K) and arginine (R) a positive 1 charge and all other amino acid residues a charge of 0. Average charge values were then calculated along a 10-amino acid long sliding window for each protein’s amino acid sequence (starting at the first residue and ending at the last complete set of 10 residues). The distribution of average charge windows for all proteins were binned into groups of X<=1, 1<X<=2, 2<X<=3, and 3<X. The median value for the average window charge across all proteins detected in the pSILAC study was 1.2. For each protein, the percentage of 10-amino acid windows with high positive charge (charge score >2) was also calculated.

Geneset enrichment analysis was conducted using the Over-Representation Analysis method and an unranked list of significantly downregulated genes from the mass spectrometry experiment. Genesets were obtained from MsigDB using the clusterProfiler package in R. For each geneset and subcategory (Hallmark, CP, GO, CGP, C6, and C1), Over-Representation Analysis was run and results pooled for visualization.

ClusterProfiler (RRID:SCR_016884): <http://dx.doi.org/10.1089/omi.2011.0118>

ORA: <https://doi.org/10.1093/bioinformatics/bth456>

Drug sensitivity cutoffs for panel of 33 CRC-derived cell lines: CRC-derived cell lines (33 total) were grouped into sensitive, intermediate, and resistant groups using empirically derived cutoffs on GI50 and Amax drug response metrics. Expression and CNV data for CRC cell lines were downloaded from the CCLE (DepMap 22Q1; https://depmap.org/). Consensus molecular subtypes (CMSs) for colorectal cancer cell lines were computed using the genes associated with each of the subtypes from Guinney et al ([1](#_ENREF_1)). Gene signature scores were computed for each subtype by taking the average z-scaled gene expression for the genes associated with those subtypes. Subtypes were assigned to cell lines based on the highest gene signature score. Chr20q11-13 CNV ratios were computed by taking the average of the CNV ratios for genes in this chromosomal segment. Genes were mapped to their chromosomal segments using the latest stable ensemble genome release containing chromosomal band annotations (GRCh37). Chromosomal segment scores were generated for each cell line by taking the average CNV ratios across genes mapping to chromosomal segments Chr20q11, Chr20q12, and Chr20q13. Cell lines having high chromosomal segment scores are indicative of amplified segments while low scores represent segment deletions. Visualizations and statistical analyses were performed using R 4.1.2 version. Packages used: tidyverse 1.3.1 (RRID:SCR_019186), data.table 1.14.2, ComplexHeatmap 2.10.0 (RRID:SCR_017270), dittoSeq 1.6.0.

**References**

1. Guinney J, Dienstmann R, Wang X, de Reynies A, Schlicker A, Soneson C*, et al.* The consensus molecular subtypes of colorectal cancer. Nat Med **2015**;21(11):1350-6 doi 10.1038/nm.3967.
